# New Experimental Treatments for Core Social Domain in Autism Spectrum Disorders

**DOI:** 10.3389/fped.2014.00061

**Published:** 2014-06-20

**Authors:** Roberto Canitano

**Affiliations:** ^1^Division of Child Neuropsychiatry, University Hospital of Siena, Siena, Italy

**Keywords:** autism spectrum disorders, experimental treatments, preclinical models, clinical trials, childhood and adolescence

## Abstract

Current therapeutics in autism spectrum disorders (ASD) only treat the associated symptoms, without addressing core social dysfunctions. A paradigm shift in research of the pathogenesis of ASD, its synaptic abnormalities and altered signaling in multiple dynamic systems, have led to new experimental treatments for treating the core social abnormalities of ASD. NMDA antagonists, especially memantine, have been introduced in clinical trials addressing glutamatergic transmission in children and adolescents with ASD. GABAergic signaling has been targeted in trials using the GABA_B_ receptor agonist arbaclofen for ASD patients with promising results. Oxytocin has been recognized as implicated in social development and affiliative behaviors. Preliminary findings from clinical trials using oxytocin in children with ASD show encouraging improvements in social cognition, but larger studies are needed. In two of the single gene disorders associated with ASD, Insulin Growth Factor (IGF-1) is a new treatment that has been tested in Rett syndrome and Phelan-McDermid syndrome (Chromosome 22 deletion syndrome). IGF-1 has been demonstrated to reverse the reduction in the number of excitatory synapses and the density of neurons that characterize these conditions in animal studies and it is being introduced as an experimental treatment. As a novel approach to verify treatment efficacy, neural processing modifications were recently evaluated by fMRI after a pivotal response training intervention. Another study of neural changes in response to treatment examined variations in EEG signaling in patients after an Early Start Denver Model (ESDM) intervention.

## Introduction

Autism spectrum disorders (ASD) are early-onset neurodevelopmental disorders characterized by major difficulties in social interaction, communication, and repetitive or restricted interests and behaviors. Autism is defined as a spectrum disorder due to the heterogeneity of clinical presentation, the degree of social impairment, intellectual ability, associated symptoms, and possible etiology. ASD are included in the diagnostic category of neurodevelopmental disorders in the Diagnostic and Statistical Manual of Mental Disorders V (1). The diagnosis of ASD is based on two major symptoms: social-communication deficits, restricted and repetitive interests, and behaviors. By definition, these symptoms must occur during the early childhood of individuals with ASD.

The currently accepted prevalence of ASD, based on consistent reports from multiple sources in different populations, is about 1% worldwide. ASDs are therefore among the most common pervasive developmental disorders and there is great concern regarding its growing incidence (2–4).

To date, the only FDA-approved treatments for ASD are the atypical antipsychotics risperidone and aripiprazole, which are mainly directed at treating the associated symptoms and not the core social dysfunctions that characterize this heterogeneous group of disorders. Treatments with these two medications have been demonstrated to reduce and attenuate irritability (e.g., tantrums, aggression, hyperactivity, and self-injurious behaviors) in children and adolescents with ASD (5–8). Improvements in social interaction and reciprocity have been observed as well, but this is probably a secondary effect of an overall reduction in maladaptive behaviors and not a primary therapeutic effect of these medications.

Targeted treatments for ASD are developed through the understanding of molecular and cellular abnormalities that guide specific interventions, hypothesizing that the wide variety of genetic variants in ASD converge in a core set of molecular pathways that mediate phenotypic expression in some identifiable core symptoms (9). Most research for new therapeutics currently uses preclinical models, such as “knockout” mice displaying specific molecular abnormalities. Genetic studies of ASD and related neurodevelopmental disorders have provided classes of potentially useful compounds. Proof-of-principle assays with agents that reversed phenotypes in mouse models have paved the way for clinical trials (10).

## Excitatory/Inhibitory Imbalance in ASD and New Treatments

An imbalance of excitatory (glutamate) to inhibitory (GABA) neurotransmission (E/I imbalance) is thought to be implicated in the pathogenesis of ASD (11–13). Excessive excitatory glutamatergic neurotransmission with a loss of inhibitory GABA transmission, as well as abnormalities in synaptic plasticity due to dysfunctions in the NMDA, AMPA, and/or GABA receptor systems, have been detected in mouse models and support this conceptualization of ASD pathogenesis (10). Pharmacological evidence emerged from mouse models with deletions in synaptic genes Fmr1, Mecp2, and Shank2, and the BTBR inbred strain, which have demonstrated favorable outcomes from treatments with glutamatergic agents, including memantine (14). After these preclinical investigations, clinical trials were carried out to test the potential role of glutamatergic and GABAergic agents in reversing core social dysfunction in ASD.

### Glutamatergic targeting in ASD

Altered glutamatergic excitatory transmission involves different receptors, including down-regulation of AMPA receptors, abnormalities of NMDA receptor-mediated plasticity, and altered metabotropic glutamate receptor subtype 5(mGluR5) signal transduction. The mGluR5 antagonists have been tested in preclinical models of autism with variable effects on social and stereotypic behaviors (15). MPEP, an antagonist of the mGluR5 receptor, reduced repetitive self-grooming in BTBR mice. BTBR is an autism mouse model with behavioral abnormalities in social interaction and communication, as well as repetitive movements and behaviors. Risperidone also reduced repetitive self-grooming in BTBR, but only at doses that induced sedation (16). GRN-259, a selective negative allosteric modulator of the mGluR5 receptor, was tested in BTBR mice. GRN-529 reduced repetitive behaviors in three cohorts of BTBR mice and it attenuated social withdrawal with an associated increase in communication, providing overall improvement in behavioral conditions (17). Treatment with the two AMPAKINE compounds CX1837 and CX1739, agents active on AMPA receptors that enhance excitatory glutamatergic action, succeeded in recovering social impairment but it did not attenuate the high levels of repetitive self-grooming in BTBR (18). In 4-week-old Balb/c mice, the NMDA receptor agonist d-cycloserine was found to improve both sociability and repetitive behaviors, emphasizing the potential role of NMDA agents in modulating the core social dysfunctions of ASD (19). Studies of Shank3 heterozygous mice detected a reduction in basal neurotransmission, reflecting reduced AMPA receptor-mediated transmission, which was targeted with insulin growth factor (IGF-1) treatment (20, 21).

Clinical trials followed the preclinical phase of investigation with glutamatergic agents. Memantine, an NMDA receptor antagonist, was tested in children and adolescents with ASD. Overall, the memantine trials conducted so far have yielded inconsistent findings due to methodological flaws, thus they did not allow for firm conclusions. No recommendations on the use of memantine or of other glutamatergic agents in ASD treatment are warranted as yet (22).

### GABAergic targeting in ASD

GABAergic signaling has been demonstrated to be implicated in E/I imbalance in ASD (23). Engrailed2 (En2) knockout mice have been proposed as a model for ASD. In this mutant, the cerebellum and hippocampus have been shown to have abnormal synaptic transmission, altered developmental processes and defective GABA transmission convergent with ASD pathways. A link between altered function of En2, deficits of GABAergic forebrain neurons, and the pathogenesis of ASD has been postulated (24, 25). In addition, social and cognitive abnormalities have been detected in En2 knockout mice, further highlighting its role in ASD. Deficits in reciprocal social interactions growing over time were found in En2 null mutants (26). Interestingly, mice lacking Mecp2 from GABA-releasing neurons demonstrated Rett syndrome and autistic features, including repetitive behaviors. In these mice, MeCP2-deficient GABAergic neurons showed a presynaptic reduction in glutamic acid decarboxylase 1 (Gad1) and glutamic acid decarboxylase 2 (Gad2) levels, and GABA immunoreactivity (27).

In clinical trials, GABA signaling has been probed with specific compounds. Arbaclofen, a GABA_B_ receptor agonist that acts upstream of mGluR5 receptor signaling and is thought to augment inhibitory neurotransmission, was investigated. Initially, a preclinical study (28) followed by a clinical trial with arbaclofen was carried out in Fragile X syndrome, the most common cause of intellectual disability and ASD (29), and favorable findings were reported regarding social domains (30). A recent open-label trial with STX209, a form of Arbaclofen, was conducted in ASD patients (31). Safety, tolerability, and efficacy were tested in ASD individuals in an 8-week open-label trial enrolling 32 children and adolescents with a score of ≥17 on the aberrant behavior checklist (ABC)-irritability subscale. STX209 was generally well tolerated. Improvements were reported on several outcome measures, including the ABC-Irritability (the primary endpoint) and the Lethargy/Social Withdrawal subscales, and the Social Responsiveness Scale. Randomized controlled trials are needed to confirm these preliminary positive findings.

### mTOR targeting in ASD

The mammalian target of rapamycin (mTOR) pathway is central to synaptic protein synthesis and it integrates inputs from different sources, including NMDA and metabotropic glutamate receptors. In addition, it is directly involved in the maintenance of the physiological synaptic E/I ratio. Abnormalities in mTOR signaling have been found in ASD (32). Activation of mTORC1 promotes the formation of the eIF4F initiation complex. Mutations in genes upstream of mTOR, as detected in tuberous sclerosis complex (TSC1/2), neurofibromatosis 1(NF1), phosphatase and tensin homolog (PTEN), or the Fragile X syndrome gene (FMR1) cause hyperactivity of the mTORC1–eIF4E pathway and lead to syndromic forms of ASDs (33, 34). Accordingly, mTOR inhibitors are first line candidates for the treatment of ASD in TSC (35). Everolimus, an mTOR inhibitor, is currently being studied in a double-blind controlled trial in children and adolescents (6–21 years old) with TSC, ASD and seizures (NCT01289912).^1^ The primary aim of this trial is improvement in cognition, while ASD symptoms attenuation and seizure frequencies are secondary objectives. Rapamycin, an mTORC1 inhibitor and immune-suppressor, was investigated in a recent preclinical study as an agent to ameliorate social behaviors and attenuate stereotypies in BTBR mice. Rapamycin was found to improve many components of sociability in the BTBR mouse, pointing to mTOR overactivation as a therapeutic target in ASD. However, no effects of rapamycin on stereotypic behaviors were detected (36).

An important regulatory action was recently described in the other component of this pathway, the downstream effectors of mTOR. Signaling molecules in the downstream of the mTOR pathway have been demonstrated to play crucial roles in ASD pathogenesis, further highlighting its role (37, 38). Furthermore, 4E-BP2 inhibits protein translation by competing with eIF4G for eIF4E binding. The deletion of the gene encoding 4E-BP2 (EIF4ebp2) leads to autistic-like behaviors in mice. It was demonstrated that the removal of 4E-BP2, or overexpression of eIF4E, enhances protein translation; increased translation of neuroligins (NLGNs) is the next step that in turn causes an increased synaptic E/I ratio, which may eventually lead to ASD phenotypes. Pharmacological inhibition of eIF4E reversed social behavior deficits in EIF4ebp2 knockout mice and restored the E/I balance (37). These findings thus established a strong link between eIF4E-dependent translational control of NLGNs, E/I balance, and the development of ASD-like behaviors. Pharmacological targeting of downstream effectors of mTOR may represent promising experimental therapeutics in ASDs.

## Single Gene Disorders with ASD and Experimental Treatments

Single gene disorders associated with high rates of comorbid ASD, such as Rett syndrome and Phelan-McDermid syndrome (PMS) have provided significant models of targeted interventions with novel experimental treatments.

### IGF-1 in Phelan-McDermid syndrome (chromosome 22q13 deletion syndrome)

The loss of a functional copy of the SHANK3 gene leads to 22q13 deletion syndrome, a complex clinical condition known as PMS. Haploinsufficiency of SHANK3 accounts for about 0.5% of cases of ASD and/or developmental delay (39). There is mounting evidence for a wider role of SHANK3 and glutamate signaling abnormalities in ASD and related conditions. IGF-1 regulates synapse formation, neurotransmitter release, and neuronal excitability via posttranslational modification of NMDA and AMPA receptors (40).

Therapeutic approaches with IGF-1 aimed at reversing deficits in SHANK3-haploinsufficiency have been carried out in mice. IGF-1 has been demonstrated to reverse the reduction in excitatory synapse numbers and density of neurons. Administration of daily intraperitoneal injections of human IGF-1 over a 2-week period was found to reverse deficits in hippocampal AMPA signaling, long-term potentiation (LTP), and motor performance in Shank3-deficient mice (41). These findings led to a current trial of IGF-1 as a treatment in PMS, which is still underway and expected to provide important clues on its role in the treatment of this ASD subtype (see text footnote 1 NCT0152590).

In a recent study, induced pluripotent stem cells (iPSCs) from individuals with PMS and autism were used to produce functional neurons. It was demonstrated that PMS neurons have reduced SHANK3 expression and major defects in excitatory, but not inhibitory, synaptic transmission. IGF-1 treatment promoted the formation of mature excitatory synapses that lack SHANK3 but contain PSD95 and NMDA receptors with fast deactivation kinetics. These findings provided evidence for a disruption in the ratio of E/I in PMS neurons, and they point to a molecular pathway that can be recruited to restore it (42).

### IGF-1 in Rett syndrome

Studies in mouse and human neuronal models of Rett syndrome also demonstrated benefits with IGF-1 administration. In 90% of cases, Rett syndrome is caused by abnormalities on the MECP2 gene, by either deletions or mutations. IGF-1, highly expressed during brain development and very important for spine maturation, has been tested to overcome MECP2 deficit. Synaptic abnormalities have been observed in Rett syndrome related to genetic defects on the MECP2 gene (43). Treatment with IGF-1 in a mouse model of Rett syndrome has been shown to increase synaptic growth and to rescue phenotype defects (44). A phase II randomized placebo-controlled trial is underway to evaluate the safety and efficacy of IGF-1 in treating ASD symptoms and respiratory/autonomic dysfunction in patients with Rett syndrome (see text footnote 1 NCT01777542).

## Intranasal Oxytocin in ASD

There has been growing interest in oxytocin as it is recognized to be implicated in social development and affiliative behaviors. Intranasal oxytocin has been shown to increase social initiative and motivation (45), as well as social cognition (46), and to reduce repetitive and restricted behaviors in ASD. Previous studies with oxytocin in ASD were conducted by single-dose administration to adults, therefore, the long-term effect of nasal oxytocin and its effect on children must still be clarified (47).

There have been only a few trials with intranasal oxytocin in children with ASD, and most were pilot studies on small numbers of patients. In one of the first double-blind studies, oxytocin nasal spray was found to improve emotion recognition in 16 male youth aged 12–19 with ASD (48). In a recent pilot study, 15 children and adolescents with ASD with verbal IQs of ≥70 participated in a trial on the safety and tolerability of intranasal oxytocin. Repeated measures of regression analysis controlled for week, dose, age, and sex were employed. The highest dose evaluated, 0.4 IU/kg, was found to be well tolerated. Over 3 months of treatment, social cognition, repetitive behaviors, and anxiety were improved with the maintenance of effect for 3 months after discontinuation of treatment (49). In an open-label study, oxytocin was administered intranasaly over a long term of 7 months to eight male youths with ASD (10–14 years of age; IQ: 20–101). Six of the eight participants had improvement in the communication and social interaction domains at ADOS-G evaluation and at *T*-scores of the CBCL; however, no statistically significant improvement was found in the ABC (50). In a double-blind study with a controlled design, a group of 38 male youths (7–16 years old) with ASD were administered 24 or 12 IU, depending on weight, of intranasal placebo or oxytocin once daily over four consecutive days. The oxytocin or placebo was administered in a dynamic social condition, i.e., during parent–child interaction training sessions. In this study, intranasal oxytocin did not significantly improve social interaction skills, however it must be mentioned that it was an untested behavioral intervention, limiting the interpretation of results (51). Collectively, these studies demonstrated mixed results as to the outcome of core social impairment, with a tendency toward an improvement in social domains. Large-scale, double-blind placebo-controlled studies are needed to confirm the role of intranasal oxytocin in core social ASD dysfunction.

A randomized, double-blind cross over fMRI trial with intranasal oxytocin was conducted in a group of 17 ASD children and adolescents (age 8–16.5 years) to evaluate neural circuit modifications hypothesized after oxytocin administration (52). The Reading the Mind in the Eyes Test (RMET), a social cognition test (53) was used to investigate the brain areas under examination, and a remarkable variation was observed 45 min after oxytocin administration. Structures and circuits within the so-called social brain, such as the dorsal and ventral striatum, premotor cortex, posterior cingulate, and posterior–superior temporal sulcus, demonstrated enhanced activity after oxytocin administration. Moreover, changes in salivary oxytocin concentrations from baseline to 30 min post administration were positively associated with increased activity in the right amygdala and orbito-frontal cortex during social vs. non-social judgments. Furthermore, it was highlighted that oxytocin enhanced the salience of social stimuli, thus positively interfering with social cognition. These changes in the relevant brain areas of interest were observed following a single oxytocin administration and further research to test the persistence of these changes over time in long-term treatments is needed.

## New Approaches to Verify Treatment Efficacy

Neural circuit modification following treatment for ASD is a novel field of research that could shed light on the mechanisms involved in neural changes and in the search for the biomarkers of treatment response (54). Functional MRI is the main instrument for a dynamic investigation of the neural substrate. A case study in two children with ASD was carried out with functional MRI performed before and after an intensive intervention of 4 months of pivotal response training (PRT). PRT is an empirically validated behavioral treatment that has widespread positive effects on communication, behavior, and social skills in young children with ASD. Neural processing modifications during a biological motion task, a form of social information, were evaluated by fMRI. Outcome measures included the evaluation of dysfunctional areas identified in children with ASD, compared to typically developing children (right amygdala, bilateral fusiform gyri, left ventrolateral prefrontal, and right posterior superotemporal sulcus). After treatment, ASD children showed increased activation in the above listed areas, indicating that neural modifications had occurred and they overlapped with the brain area activations of their typically developing peers (55).

Another study, investigating neural changes in response to treatment, examined variations in EEG signaling after an intervention with the Early Start Denver Model (ESDM) in a group of 48 children with ASD, aged 18–30 months. Children with ASD were randomized into two groups; one received an ESDM intervention for 2 years and the other received only their previous treatment in the community. A typically developing group of children served as controls. Event related potentials (ERP) and EEG activity (spectral power) were detected during the presentation of faces versus objects. The ESDM group demonstrated a better outcome in ASD symptoms, IQ, language, and adaptive behavior than the others. Increased cortical activation, i.e., a reduction in alfa power and increased theta band, was detected in this group of children as well as in typically developing children when viewing faces, in contrast to the community intervention group that showed the opposite pattern with greater cortical activation when viewing objects. The limitation of this research was that it lacked a baseline evaluation (56).

## Conclusion and Future Directions

Currently available medications mostly act upon selective symptoms of ASD, possibly offering a chance to further explore the specific mechanisms and circuits involved in etiological factors. Synaptic or circuit mechanisms associated with circumscribed aspects of ASD, e.g., social and communication domains, need to be thoroughly evaluated at the molecular and synaptic levels and possibly separated from the other symptoms/domains. Core mechanisms that cover distinct fractions of ASD would parallel the concept of core symptoms in ASD, based on the hypothesis that a group of protein factors converges on common pathways to be targeted (13). The next generation of animal models carrying human mutations will be of the utmost importance in uncovering the neural and molecular bases of ASD and to pave the way for clinical trials of treatments for core social domains. A homogeneous group of ASD individuals with phenotype and synaptic defects should be enrolled in pharmacological trials to test the efficacy and the safety of specific compounds. These trials would measure outcomes in the core social domain of ASD and also identify specific biomarkers for a wider perspective of treatment outcomes. Investigations of dynamic brain changes in ASD patients, using fMRI after treatments, would be extremely valuable in order to shed light on the neural underpinnings of core social impairment in these individuals.

## Conflict of Interest Statement

The author declares that the research was conducted in the absence of any commercial or financial relationships that could be construed as a potential conflict of interest.

## Footnotes

^1^http://clinicaltrials.gov
